# Transcription Repressor Hes1 Contributes to Neuropathic Pain Development by Modifying CDK9/RNAPII-Dependent Spinal *mGluR5 *Transcription

**DOI:** 10.3390/ijms20174177

**Published:** 2019-08-26

**Authors:** Ming-Chun Hsieh, Hsien-Yu Peng, Yu-Cheng Ho, Cheng-Yuan Lai, Jen-Kun Cheng, Gin-Den Chen, Tzer-Bin Lin

**Affiliations:** 1 Department of Medicine, Mackay Medical College, New Taipei 25244, Taiwan; 2 Department of Anesthesiology, Mackay Memorial Hospital, Taipei 10449, Taiwan; 3 Department of Obstetrics and Gynecology, Chung-Shan Medical University Hospital, Chung-Shan Medical University, Taichung 40201, Taiwan; 4 Department of Physiology, School of Medicine, College of Medicine, Taipei Medical University, Taipei 11031, Taiwan; 5 Graduate Institute of Basic Medical Science, China Medical University, Taichung 40402, Taiwan; 6 Department of Biotechnology, College of Medical and Health Science, Asia University, Taichung 41354, Taiwan; 7 Cell Physiology and Molecular Image Research Center, Wan Fang Hospital, Taipei Medical University, Taipei 11689, Taiwan

**Keywords:** neuropathic pain, dorsal horn, Hes1, CDK9, RNAPII

## Abstract

Diverse transcriptional controls in the dorsal horn have been observed in pain hypersensitivity. However, the understanding of the exact causes and mechanisms of neuropathic pain development is still fragmentary. Here, the results demonstrated nerve injury decreased the expression of spinal hairy and enhancer of split 1 (Hes1), a transcriptional repressor, and enhanced metabotropic glutamate receptor subtype 5 (mGluR5) transcription/expression, which was accompanied with behavioral allodynia. Moreover, nerve injury decreased Hes1 levels and reciprocally increased cyclin dependent kinase-9 (CDK9) levels and recruited CDK9 to phosphorylate RNA polymerase II (RNAPII) in the promoter fragments of mGluR5, thereby enhancing mGluR5 transcription/expression in the dorsal horn. These effects were also induced by intrathecally administering naïve rats with Hes1 small interfering RNA (siRNA). Conversely, Hes1 overexpression using intrathecal lentiviral vectors in nerve injury rats produced reversal of pain behavior and reversed protein expressions, phosphorylation, and coupling to the promoter segments in the dorsal horn. Collectively, the results in this study indicated nerve injury diminishes spinal Hes1-dependent suppression of CDK9-dependent RNAPII phosphorylation on the mGluR5 promoter that possibly enhances mGluR5 transcription/expression for neuropathic pain development.

## 1. Introduction

In contrast to most mammalian basic helix-loop-helix (bHLH) transcription factors site-specifically activating gene expression [1], hairy and enhancer of split (Hes) is a unique bHLH [2] that represses genes transcription [3,4] to regulate neural morphology and functions [5]. Among the Hes1-7 families, Hes1 is recognized as a regulator for the transcription of synaptic proteins and thereby crucially impacts synaptic transmission and plasticity development [6]. Down-regulated Hes1 expression increases synapses integration in rats’ dentate gyrus accompanied with progression of memory-associated plasticity [7], and conversely, reagent-induced Hes1 expression in the rat hippocampus results in learning and memory deficits [8]. Nevertheless, though mounting evidence has shown that spinal pain-associated plasticity relies on molecular processes mediating learning and memory in brain regions [9,10], the potential involvement of Hes1-suppressed genes transcription in the spinal plasticity underlying pain hypersensitivity has yet to be established.

Cyclin dependent kinase-9 (CDK9), an elongation factor [11], phosphorylates RNA polymerase II (RNAPII) to initiate transcription elongation of genes [12]. CDK/RNAPII-dependent elongation persuades gene transcription, mediating the development of plasticity, as evidenced by the depletion of specific CDK isoforms, which induces heterochromatin enrichment that results in learning defects in *Drosophila* [13], and the stalling of RNAPII at specific gene promoters of tegmentum neurons incites plasticity underlying morphine rewards in rats [14]. Notably, in a subset of genes, Hes1 suppresses transcription elongation by hindering the recruitment of CDK9 and the following RNAPII phosphorylation [15]. One of our publications recently linked CDK9-dependent RNAPII phosphorylation to the transcription of pain plasticity-associated genes [16]; Hes1 may contribute to pain by impacting the CDK9/RNAPII-initiated transcription of genes essential for spinal plasticity.

In addition to widely mediating physiological neurotransmission [17], spinal glutamatergic synapses also crucially underlie the pathophysiological machinery of pain hypersensitivity [18]. By focusing on the dorsal horn, the first relaying point for pain neurotransmission, our laboratory has linked metabotropic glutamate receptor subtype 5 (mGluR5), a member of the glutamatergic receptor family, to plasticity underlying neuropathic pain development not only because experimental neuropathic injury upregulated mGluR5 expression in the dorsal horn [19], but also because enhanced mGluR5 transcription in rodent dorsal horn neurons provoked allodynia, a pain sensation caused by innocuous stimulation that is a clinical sign of neuropathic pain [20]. Since Hes1 represses transcription of glutamatergic receptors and thereby reduces synaptic efficacy in cortical neurons [6], it is hypothesized that neuropathic injury-induced pain hypersensitivity involves impedance of spinal Hes1-suppressed mGluR5 transcription by prompting interactions between CDK9/RNAPII and mGluR5 promoters that enhance mGluR5 transcription. Results in this study provided a molecular, histological, behavioral, and genetic basis supporting the above hypothesis, and additionally revealed that reciprocal Hes1-CDK9 expression regulates RNAPII phosphorylation on promoter loci that impact spinal mGluR5 transcription, mediating neuropathic pain.

## 2. Results

### 2.1. SNL Decreased Spinal Hes1 and Provoked Behavioral Allodynia

**Figure 1:**
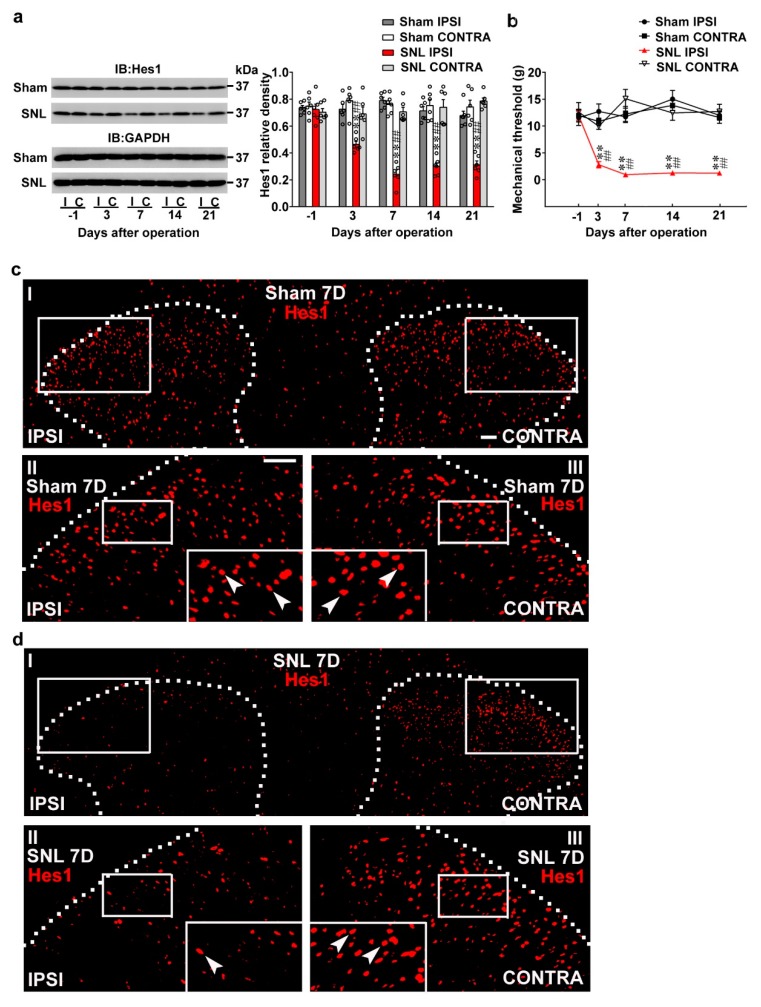
Nerve injury reduced spinal Hes1 expression accompanied with behavioral allodynia. (**a**) Representative Western blot and statistical analyses (normalized to GAPDH) demonstrating, when compared with the sham operation (Sham), that spinal nerve ligation (SNL) decreased the expression of Hes1 in the ipsilateral (I and IPSI) but not the contralateral (C and CONTRA) dorsal horn at days 3, 7, 14, and 21 after surgery. IB, Immunoblotting. Two-way ANOVA with repeated measures over time, treatment, F (3,20) = 116.6, *p* < 0.0001; time, F (4,80) = 6.258, *p* = 0.0002; treatment × time, F(12,80) = 9.003, *p* < 0.0011. ** *p* < 0.01 vs. Sham IPSI. ## *p* < 0.01 vs. SNL day –1. *n* = 6. (**b**) Von Frey test demonstrating, when compared with the sham operation, that SNL decreased the tactile withdrawal threshold of the ipsilateral but not the contralateral hind-paw at days 3, 7, 14, and 21 after surgery. Two-way ANOVA with repeated measures over time, treatment, F (3,24) = 59.37, *p* < 0.0001; time, F(4,96) = 4.514, *p* = 0.0022; treatment × time, F(12,96) = 6.427, *p* < 0.0001. ** *p* < 0.01 vs. Sham IPSI. ## *p* < 0.01 vs. SNL day –1. *n* = 7. (**c**,**d**) Immunofluorescence images showing, when compared with the sham operation (**c**), that SNL (**d**) decreased the number and distribution of Hes1-positive neurons (red) in the ipsilateral dorsal horn (left) of spinal cord slice harvested at day 7 (7D) after surgery. Dashed lines indicate the margin of the dorsal horn. The lower images (II, III) are magnifications of the upper images (I); and the inset images at the bottom of the lower image are further amplified images, with arrows marking the Hes1-positive neurons. Scale bar = 50 μm; Thickness = 30 μm.

To characterize the role of spinal Hes1 in neuropathic pain, the expression profile of Hes1 in the dorsal horn following spinal nerve ligation (SNL), a procedure mimicking neuropathic injury [21,22], was initially examined. In dorsal horn samples ipsilateral to the injury, Western blot analyses revealed SNL gradually reduced the Hes1 level, which began to fall at day 3, plateaued at day 7, and was maintained at a relatively constant level until days 14 and 21 after operation (Figure 1a; 0.46 ± 0.02, 0.24 ± 0.03, 0.30 ± 0.02, and 0.31 ± 0.02). Moreover, withdrawal threshold data collected by testing animals’ responses to von Frey hair demonstrated SNL induced tactile allodynia as evidenced by decrements in the mechanical threshold of rats’ ipsilateral hind paw, with a time course matching the spinal Hes1 diminishing (Figure 1b; 2.79 ± 0.58, 0.93 ± 0.13, 1.25 ± 0.12, and 1.24 ± 0.24 g). To further strengthen the role of Hes1 in the spinal machinery mediating the development of neuropathic pain, spinal slice dissected at day 7 after operation (a time-point at which SNL animals displayed maximal behavioral allodynia) were labeled with Hes1-specific antibody. Images in (Figure 1c,d) illustrate SNL decreased Hes1-positive neurons in the ipsilateral, but not contralateral, dorsal horn compared with the sham operation. These results indicate neuropathic injury provokes behavioral allodynia accompanied with decreased Hes1 in the ipsilateral dorsal horn.

### 2.2. Knockdown of Spinal Hes1 Expression Provokes Tactile Allodynia

**Figure 2:**
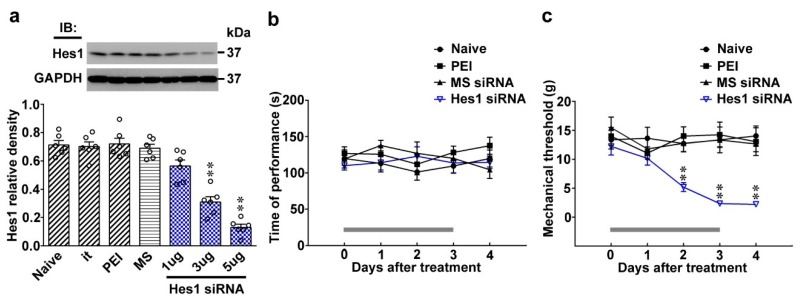
Focal knockdown of spinal Hes1 expression elicits no motor deficits but provokes behavioral allodynia. (**a**) Representative Western blot and statistical analyses (normalized to GAPDH) showing the Hes1 levels in the dorsal horn dissected from naïve animals (Naïve) were not affected by implantation of an intrathecal catheter alone (it) or spinal administration with polyethylenimine (PEI, 10 μL; daily for 4 days) or missense siRNA (MS siRNA, 5 μg, 10 μL; daily for 4 days) but was dose-dependently decreased by intrathecal administration with Hes1 siRNA (Hes1 siRNA; 1, 3, and 5 μg; 10 μL; daily for 4 days) at day 4 after the start of treatments. IB, Immunoblotting. One-way ANOVA, post-hoc Tukey test, F (6,35) = 47.9, *p* < 0.0001. ** *p* < 0.01 vs. Naïve. *n* = 6. (**b**) Rota-rod test demonstrating no statistical difference in the motor performance among naïve animals as well as naïve animals administered with polyethylenimine, missense siRNA, or Hes1 siRNA (5 μg, 10 μL). The gray bar at the bottom indicates the duration of the reagents’ administration. Two-way ANOVA with repeated measures over time, treatment, F (3,24) = 1.170, *p* = 0.3419; time, F (4,96) = 0.2094, *p* = 0.9327; treatment × time, F(12,96) = 0.8289, *p* = 0.6205. *n* = 7. (**c**) Results of the von Frey test demonstrating that administration to naïve rats with Hes1 siRNA (5 μg, 10 μL), but not polyethylenimine, or missense siRNA decreased the withdrawal threshold of the hind paw at days 2, 3, and 4 after the injection. The gray bar at the bottom indicates the duration of the reagents’ administration. Two-way ANOVA with repeated measures over time, treatment, F (3,24) = 40.16, *p* < 0.0001; time, F (4,96) = 2.481, *p* = 0.0489; treatment × time, F (12,96) = 2.226, *p* = 0.0160. ** *p* < 0.01 vs. Naïve. *n* = 7.

Based on above observations, the involvement of spinal Hes1 in allodynia was further tested by establishing a rat model in which spinal Hes1 expression was focally knocked down by daily intrathecal administration of antisense small interfering RNA (siRNA) targeted to the Hes1 mRNA. The siRNA protocol efficiently trimmed down spinal Hes1 expression without provoking any observable motor deficits in naïve animals as evidenced by Hes1 siRNA (1, 3, and 5 μg; 10 μL) dose-dependently reducing Hes1 levels in dorsal horn samples (Figure 2a; 0.56 ± 0.04, 0.30 ± 0.03, and 0.13 ± 0.02), but did not exhibit effects on the performance time measured by the rota-rod test (Figure 2b). Intriguingly, knockdown of spinal Hes1 expression provoked allodynia in naïve animals as Hes1 siRNA (5 and 10 μL) decreased the mechanical threshold at days 2, 3, and 4 after treatment (Figure 2c; 5.19 ± 0.75, 2.35 ± 0.43, and 2.22 ± 0.42 g). These data provide a genetic basis supporting the notion that down-regulated Hes1 expression is an essential factor for the spinal machinery underlying the development of tactile allodynia.

### 2.3. Spinal Hes1 Overexpression Ameliorates SNL-Provoked Allodynia

**Figure 3:**
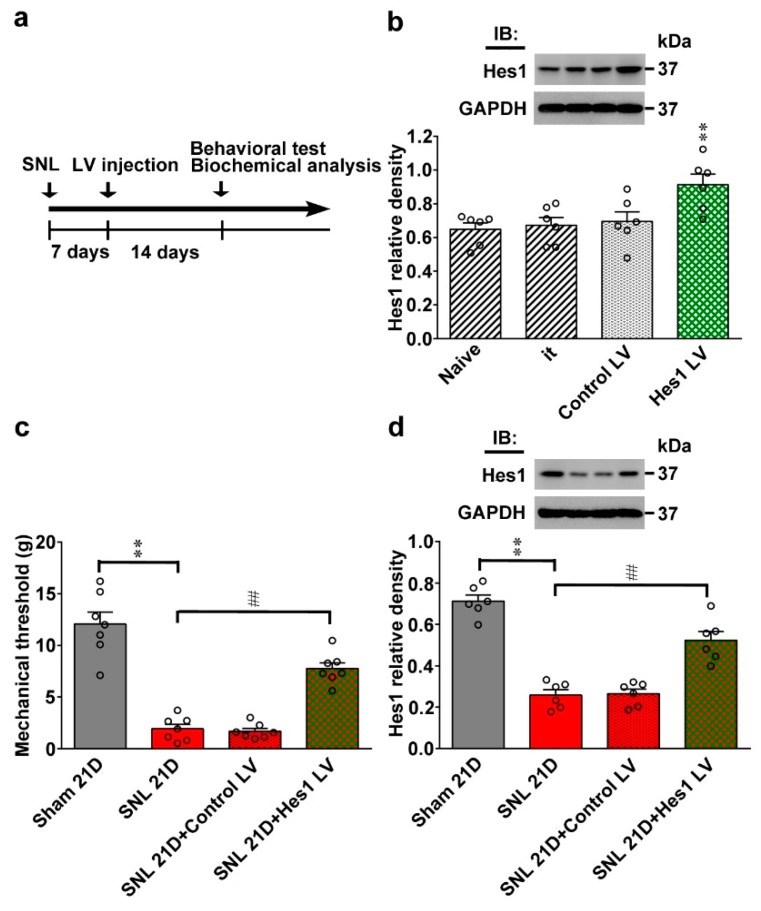
Lentiviral vector-mediated Hes1 expression reversed neuropathic allodynia. (**a**) Experimental timeline of spinal nerve ligation (SNL), lentiviral vector (LV) transfection, as well as behavioral test and biochemical analyses. Seven days after operation, lentivirus vector was spinally injected to animals, and the behavioral test or biochemical analyses were carried out 14 days after transfection. (**b**) Representative western blot and statistical analyses were performed (normalized to GAPDH). Transfecting naïve animals (Naïve) with the Hes1-encoding vector (Hes1 LV), but not with the control vector (Control LV) or implantation of the intrathecal catheter itself (it), increased the abundance of Hes1 in the dorsal horn sample. IB, immunoblotting. One-way ANOVA, post-hoc Tukey test, F(3,20) = 5.516, *p* = 0.0063. ** *p* < 0.01 vs. Naïve. *n* = 6. (**c**) Results of the von Frey test demonstrating that transfecting SNL animals with Hes1-encoding vector (SNL 21D + Hes1 LV), but not the control vector (SNL 21D + Control LV), ameliorated the SNL-decreased withdrawal threshold in the ipsilateral hind paw tested at day 21 after operation. One-way ANOVA, post-hoc Tukey test, F (3,24) = 51.08, *p* < 0.0001. ** *p* < 0.01 vs. Sham 21D. ## *p* < 0.01 vs. SNL 21D. *n* = 7. (**d**) Representative western blot and statistical analyses (normalized to GAPDH) showing that transfection of SNL animals with Hes1-encoding vector (SNL 21D + Hes1 LV) but not the control vector (SNL 21D + Control LV) reversed the SNL-decreased Hes1 expression in the ipsilateral dorsal horn sample dissected at day 21 after operation. One-way ANOVA, post-hoc Tukey test, F (3,20) = 48.96, *p* < 0.0001. ** *p* < 0.01 vs. Sham 21D. ## *p* < 0.01 vs. SNL 21D. *n* = 6.

After identifying that the role of reduced Hes1 expression in spinal mechanisms mediates the development of neuropathic allodynia, whether an enhancement of spinal Hes1 levels is sufficient to ameliorate behavioral allodynia caused by neuropathic injury was tested next. For this purpose, we intrathecally injected animals with lentiviral vectors encoding the rat Hes1 protein (Hes1 vector) or encoding GFP alone (control vector) on day 7 after SNL, and performed behavior and protein analyses on day 14 after vector injection (Figure 3a). Western blot analyses confirmed the efficacy and specificity of our protocol as gene transfer enhanced Hes1 expression in the dorsal horn of naïve rats (Figure 3b; 0.91 ± 0.06). Moreover, administration of Hes1 vector to SNL rats ameliorated the established allodynia and reversed the associated Hes1 down-regulation in the dorsal horn (Figure 3c, d; 7.75 ± 0.57 and 0.52 ± 0.04 g), implying focal Hes1 overexpression in the spinal cord is sufficient to ameliorate neuropathic injury-induced behavioral allodynia.

### 2.4. SNL Diminishes Hes1-Dependent Suppression of Spinal mGluR5 Transcription

**Figure 4:**
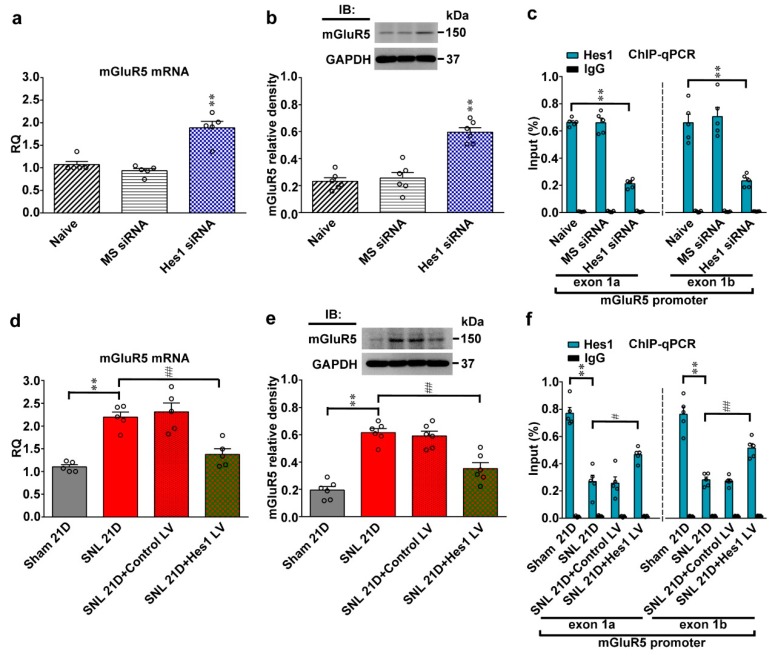
Neuropathic injury diminishes Hes1-repressed spinal mGluR5 transcription. (**a**,**b**) RT-PCR and western blot analyses (normalized to GAPDH) demonstrating that the mRNA and protein levels of mGluR5 in dorsal horn samples were increased by administering naïve animals (Naive) with Hes1 siRNA but not with missense siRNA (Hes1 siRNA and MS siRNA, respectively. 5 μg, 10 μL; it, daily for 4 days). IB, immunoblotting. mRNA, one-way ANOVA, post-hoc Tukey test, F (2,12) = 27.91, *p* < 0.0001. protein, one-way ANOVA, post-hoc Tukey test, F (2,15) = 34.80, *p* < 0.0001. ** *p* < 0.01 vs. Naïve. mRNA, *n* = 5. protein, *n* = 6. (**c**) ChIP-qPCR assay of dorsal horn samples demonstrating that administering naïve animals with Hes1 siRNA but not with missense siRNA (Hes1 siRNA and MS siRNA, respectively. 5 μg, 10 μL; it, daily for 4 days) decreased amounts of Hes1 antibody-precipitated exon 1a and exon 1b promoter fragments of mGluR5. ** *p* < 0.01 vs. Naïve. *n* = 5. Hes1, exon 1a, one-way ANOVA, post-hoc Tukey test, F (2,12) = 131.7, *p* < 0.0001. Hes1, exon 1b, one-way ANOVA, post-hoc Tukey test, F (2,12) = 22.70, *p* < 0.0001. (**d**,**e**) RT-PCR and western blot analyses of the ipsilateral dorsal horn samples dissected at day 21 after operation. When compared with the sham operation (Sham 21D), spinal nerve ligation (SNL 21D) increased mRNA and protein levels of mGluR5 that were both reversed by intrathecal administration to SNL animals with Hes1-encoding vector (SNL 21D + Hes1 LV) but not the control vector (SNL 21D + Control LV). mRNA, one-way ANOVA, post-hoc Tukey test, F(3,16) = 20.75, *p* < 0.0001. protein, one-way ANOVA, post-hoc Tukey test, F (3,20) = 34.70, *p* < 0.0001. ** *p* < 0.01 vs. Sham 21D. ## *p* < 0.01 vs. SNL 21D. mRNA, *n* = 5. protein, *n* = 6. (**f**) ChIP-qPCR assay of dorsal horn samples dissected at day 21 post operation. When compared with the sham operation, SNL decreased the amounts of Hes1 antibody-precipitated exon 1a and exon 1b promoter fragments of mGluR5; effects that were both reversed by transfecting animals with Hes1-encoding vector (SNL 21D + Hes1 LV) but not the control vector (SNL 21D + Control LV). Hes1, exon 1a, one-way ANOVA, post-hoc Tukey test, F (3,16) = 34.72, *p* < 0.0001. Hes1, exon 1b, one-way ANOVA, post-hoc Tukey test, F (3,16) = 45.74, *p* < 0.0001. ** *p* < 0.01 vs. Sham 21D. # *p* < 0.05, ## *p* < 0.01 vs. SNL 21D. *n* = 5.

By modifying the promoter activity of genes regulating the expression of glutamatergic receptors [6], Hes1 plays as a transcription repressor to impact neuron function [5]. Notably, pain hypersensitivity in diabetic rats involves modification of gene transcription of mGluR5 [23], a molecule our laboratory has recently shown to mediate the spinal plasticity underlying the development of neuropathic allodynia [19,20]. Next, whether SNL-induced allodynia involves diminished Hes1-dependent repression of spinal mGluR5 transcription was tested first by administration of Hes1 siRNA (5 μg, 10 μL; i.t.) to naïve animals. Results of RT-PCR and Western blotting demonstrated focal knockdown of spinal Hes1 expression markedly increased mRNA and protein expressions of mGluR5 in dorsal horn samples (Figure 4a,b; mRNA, 1.88 ± 0.14; protein, 0.59 ± 0.03). Next, whether the increments in mGluR5 expression could be attributed directly to reduced Hes1 binding to mGluR5 promoter loci was tested. Chromatin immunoprecipitation (ChIP)-qPCR analyses of dorsal horn samples revealed the amount of Hes1 antibody-recognized mGluR5 promoters (i.e., exon 1a and exon 1b) was significantly decreased by Hes1 siRNA (5 μg, 10 μL; Figure 4c; exon 1a, 0.21 ± 0.01; exon 1b, 0.23 ± 0.02). Conversely, SNL upregulated both mRNA and protein expressions of mGluR5 but decreased the amount of Hes1 antibody-precipitated mGluR5 promoters (exon 1a and 1b) in dorsal horn samples (mRNA, 2.19 ± 0.11; protein, 0.61 ± 0.02; exon 1a, 0.27 ± 0.04; exon 1b, 0.28 ± 0.02); these effects were all ameliorated by intrathecal injection of the Hes1 vector (Figure 4d–f; mRNA, 1.37 ± 0.12; protein, 0.35 ± 0.04; exon 1a 0.46 ± 0.02; exon 1b, 0.51 ± 0.03). Together, these findings suggested that by impeding Hes1-dependent repression on mGluR5 transcription, neuropathic insults enhance mGluR5 expression in the dorsal horn.

### 2.5. SNL Diminishes Hes1-Dependent Suppression of Spinal CDK9/pRNAPII-Mediated mGluR5 Transcription

**Figure 5:**
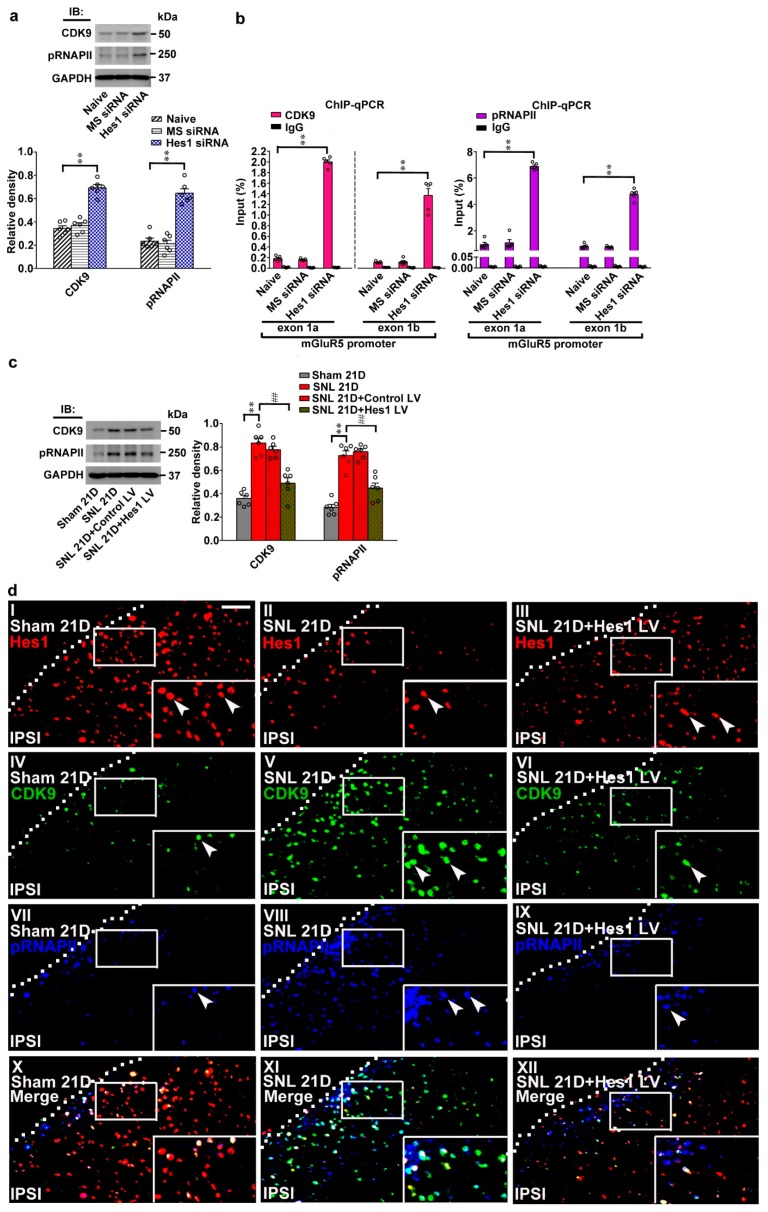

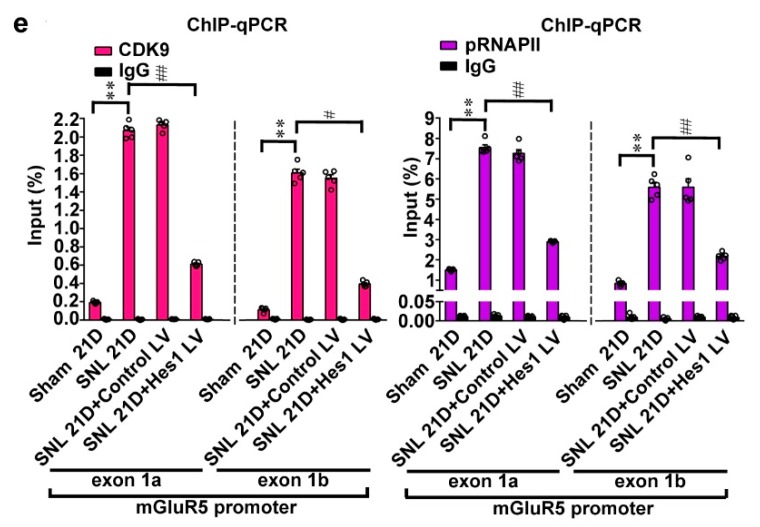
Neuropathic injury impedes Hes1-suppressed CDK9 recruitment and RNAPII phosphorylation on mGluR5 promoters. (**a**) Representative western blot and statistical analyses (normalized to GAPDH) of dorsal horn samples demonstrating the levels of CDK9 and phosphorylated RNAPII (pRNAPII) were increased by spinally administering naïve animals (Naïve) with Hes1-siRNA but not with missense siRNA (Hes1 siRNA and MS siRNA, respectively, 5 μg, 10 μL; daily for 4 days). IB, immunoblotting. CDK9, one-way ANOVA, post-hoc Tukey test, F (2,15) = 64.17, *p* < 0.0001. pRNAPII, one-way ANOVA, post-hoc Tukey test, F (2,15) = 59.83, *p* < 0.0001. ** *p* < 0.01 vs. Naïve. *n* = 6. (**b**) ChIP-qPCR assay of dorsal horn samples demonstrating that administering naïve animals with Hes1 siRNA, but not missense siRNA (Hes1 siRNA and MS siRNA, respectively. 5 μg, 10 μL; daily for 4 days), increased amounts of both CDK9 and pRNAPII antibody-precipitated exon 1a and exon 1b promoter fragments of mGluR5. CDK9, exon 1a, one-way ANOVA, post-hoc Tukey test, F (2,12) = 1494, *p* < 0.0001. CDK9, exon 1b, one-way ANOVA, post-hoc Tukey test, F(2,12) = 88.97, *p* < 0.0001. pRNAPII, exon 1a, one-way ANOVA, post-hoc Tukey test, F (2,12) = 358.4, *p* < 0.0001. pRNAPII, exon 1b, one-way ANOVA, post-hoc Tukey test, F (2,12) = 373.5, *p* < 0.0001. ** *p* < 0.01 vs. Naïve. *n* = 5. (**c**) Representative western blot and statistical analyses of dorsal horn samples dissected at day 21 after operation. When compared with the sham operation (Sham 21D), spinal nerve ligation (SNL 21D) increased spinal CDK9 and pRNAPII levels; that were both reversed by administering SNL animals with Hes1-encoding vector (SNL 21D + Hes1 LV) but not the control vector (SNL 21D + Control LV). CDK9, one-way ANOVA, post-hoc Tukey test, F (3,20) = 38.06, *p* < 0.0001. pRNAPII, one-way ANOVA, post-hoc Tukey test, F (3,20) = 43.90, *p* < 0.0001. ** *p* < 0.01 vs. Sham 21D. ## *p* < 0.01 vs. SNL 21D. *n* = 6. (**d**) Immunofluorescence images of spinal slices dissected at day 21 post operation. Compared with the sham operation (left), SNL (middle) decreased the number and distribution of the Hes1-positive (red) neurons but increased that of the CDK9-postive (green) and pRNAPII-postive (blue) neurons in the dorsal horn; these effects were all reversed by transfecting SNL animals with Hes1-encoding vector (right). Dashed lines indicate the margin of the dorsal horn. The inset images at the bottom are amplifications of the upper marked area, with arrows indicating the immunopositive neurons. Scale bar = 50 μm; Thickness = 30 μm. (**e**) ChIP-qPCR assay of dorsal horn samples dissected at day 21 post operation. When compared with the sham operation, SNL increased the amounts of CDK9 and pRNAPII antibodies-precipitated exon 1a and exon 1b promoter fragments of mGluR5; effects were both reversed by transfecting SNL animals with Hes1-encoding vector but not the control vector. CDK9, exon 1a, one-way ANOVA, post-hoc Tukey test, F (3,16) = 1812, *p* < 0.0001. CDK9, exon 1b, one-way ANOVA, post-hoc Tukey test, F (3,16) = 675.6, *p* < 0.0001. pRNAPII, exon 1a, one-way ANOVA, post-hoc Tukey test, F (3,16) = 638.7 *p* < 0.0001. pRNAPII, exon 1b, one-way ANOVA, post-hoc Tukey test, F (3,16) = 103.0 *p* < 0.0001. ** *p* < 0.01 vs. Sham 21D. # *p* < 0.05, ## *p* < 0.01 vs. SNL 21D. *n* = 5.

As Hes1 is shown to hamper transcription elongation by hindering gene recruitment of the CDK9 [11] and subsequent RNA polymerase II phosphorylation [15], whether diminished Hes1-repressed mGluR5 transcription caused by SNL is mediated by modifying CDK9 coupling and RNAPII phosphorylation on mGluR5 promoters was tested next. First, administering naïve animals with Hes1 siRNA (5 μg, 10 μL) upregulated levels of CDK9 and phosphorylated RNA polymerase II (pRNAPII) (Figure 5a; 0.69 ± 0.02 and 0.64 ± 0.03) as well as increased the amounts CDK9 and pRNAPII antibodies-precipitated mGluR5 promoter segments (exon 1a and exon 1b) (Figure 5b; CDK9, 1.99 ± 0.04 and 1.36 ± 0.13; pRNAPII, 6.88 ± 0.11 and 4.75 ± 0.18) in the dorsal horn. On the other hand, SNL also increased the amounts of CDK9 and pRNAPII proteins in the dorsal horn samples (0.83 ± 0.04 and 0.72 ± 0.04); these effects were attenuated by intrathecal injection of the Hes1 vector (Figure 5c; 0.49 ± 0.04 and 0.44 ± 0.04). Consistent with these results, immunofluorescence images demonstrated that SNL decreased Hes1-positive immunoreactivity, but increased CDK9-positive and pRNAPII-positive immunoreactivity in the dorsal horn of spinal slices; these effects were attenuated by intrathecal injection of the Hes1 vector (Figure 5d). Furthermore, the Hes1 vector reversed the enhancement of CDK9 antibody- and pRNAPII antibody-precipitated mGluR5 promoters (exon 1a and exon 1b) in the dorsal horn samples caused by SNL (Figure 5e; CDK9, from 2.06 ± 0.03 to 0.61 ± 0.01 and from 1.60 ± 0.04 to 0.39 ± 0.01; pRNAPII, from 7.54 ± 0.14 to 2.89 ± 0.01 and from 5.59 ± 0.22 to 2.17 ± 0.08). Collectively, these results suggest neuropathic injury diminishes Hes1-suppression of mGluR5 transcription mediated by enhanced CDK9 coupling and RNAPII phosphorylation on mGluR5 promoters.

### 2.6. Reciprocal Spinal Hes1-CDK9 Expression Underlies Neuropathic Allodynia

**Figure 6:**
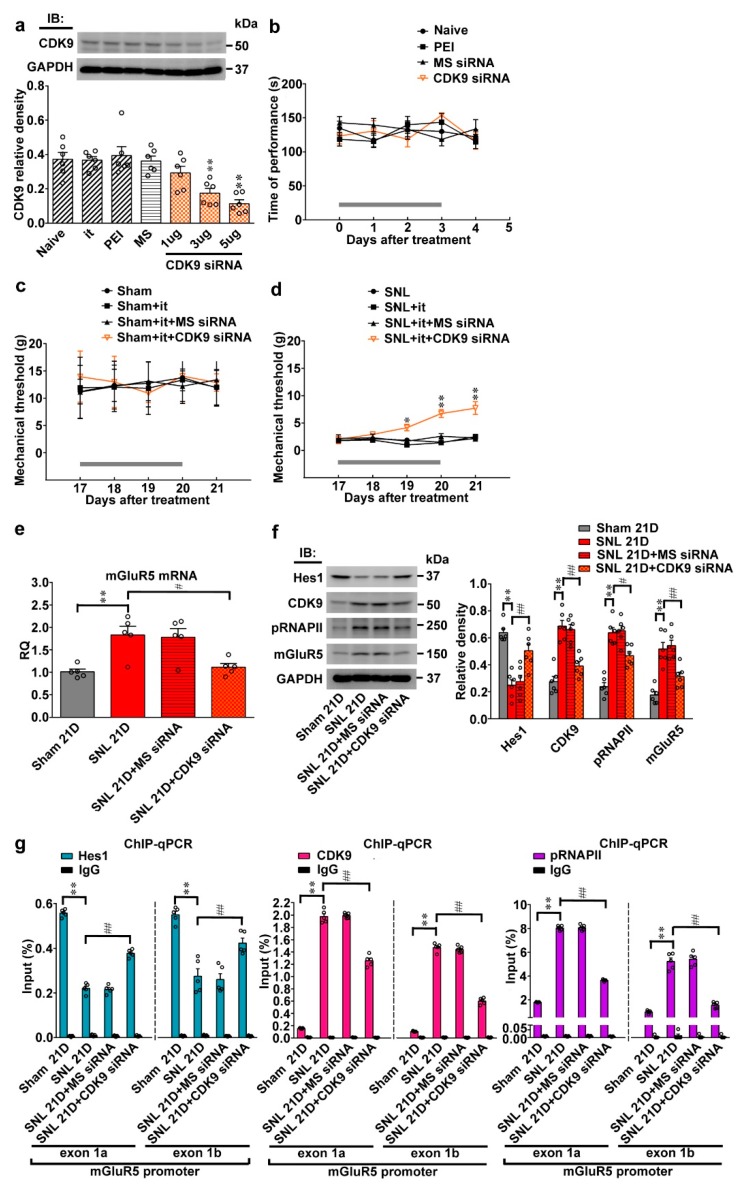

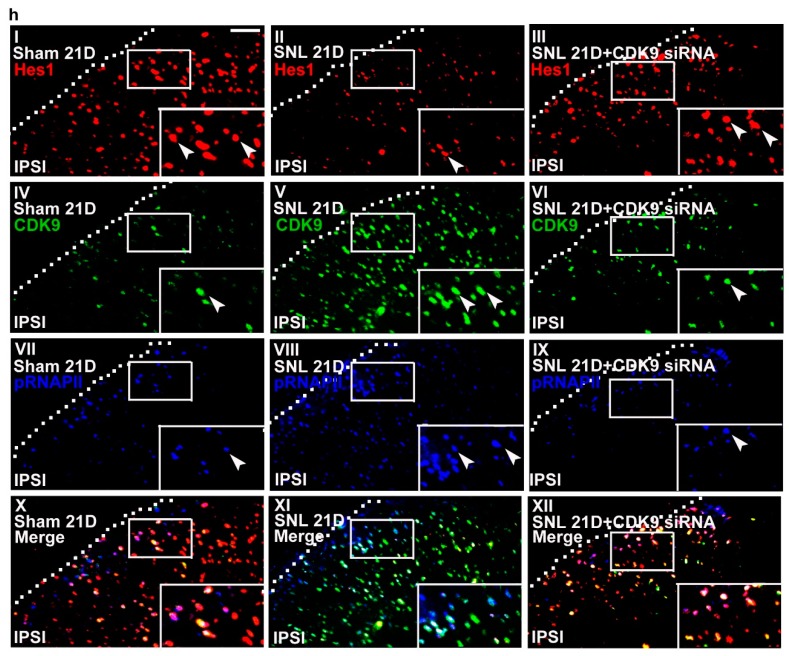
Neuropathic injury induces allodynia with reciprocal changes in spinal Hes1-CDK9 levels. (**a**) Representative Western blot and statistical analyses (normalized to GAPDH) of the dorsal horn sample dissected from naïve animals (Naïve) at day 4 after the start of treatment. The abundance of CDK9 was not affected by implantation of an intrathecal catheter alone (it) or spinal administration of polyethylenimine (PEI, 10 μL; daily for 4 days) or missense siRNA (MS siRNA, 5 μg, 10 μL; daily for 4 days) but was dose-dependently decreased by intrathecal administration of CDK9 siRNA (CDK9 siRNA; 1, 3, and 5 μg; 10 μL; daily for 4 days). IB, Immunoblotting. One-way ANOVA, post-hoc Tukey test, F (6,35) = 10.16, *p* < 0.0001. ** *p* < 0.01 vs. Naïve. *n* = 6. (**b**) Rota-rod test demonstrating no statistical difference in the motor performance among naïve animals as well as naïve animals administered with polyethylenimine, missense siRNA, or CDK9 siRNA (5 μg, 10 μL). The gray bar at the bottom indicates the duration of the reagents’ administration. *n* = 7. Two-way ANOVA with repeated measures over time, treatment, F (3,24) = 0.4792, *p* = 0.6997; time, F (4,96) = 0.2094, *p* = 0.9351; treatment × time, F (12,96) = 1.123, *p* = 0.3510. (**c**,**d**) Von Frey test demonstrating that while it exhibited no effect on the sham-operated group, spinal administration of CDK9 siRNA (5 μg, 10 μL), but not missense siRNA or polyethylenimine, increased the withdrawal threshold of the ipsilateral hind paw of SNL rats at days 19, 20, and 21 after operation. The gray bar at the bottom indicates the duration of the reagents’ administration. * *p* < 0.05, ** *p* < 0.01 vs. SNL. *n* = 7. Sham, Two-way ANOVA with repeated measures over time, treatment, F (3,24) = 0.2648, *p* = 0.8501; time, F (4,96) = 0.4465, *p* = 0.7747; treatment × time, F(12,96) = 0.3796, *p* = 0.9679. SNL, Two-way ANOVA with repeated measures over time, treatment, F (3,24) = 32.28, *p* < 0.0001; time, F(4,96) = 7.267, *p* < 0.0001; treatment × time, F(12,96) = 5.291, *p* < 0.0001. (**e**,**f**) RT-PCR and western blot analyses (normalized to GAPDH) of ipsilateral dorsal horn samples dissected at day 21 post operation. When compared with the sham operation (Sham 21D), SNL (SNL 21D) increased the abundance of mGluR5 mRNA as well as CDK9, phosphorylated RNAPII (pRNAPII), and mGluR5 protein but decreased that of Hes1 protein. These effects were all reversed by administering SNL animals with CDK9 siRNA (SNL 21D + CDK9 siRNA) but not the missense siRNA (SNL 21D + MS siRNA). mGluR5 mRNA, one-way ANOVA, post-hoc Tukey test, F (3,16) = 8.639, *p* = 0.0012. Hes1, one-way ANOVA, post-hoc Tukey test, F (3,20) = 19.59, *p* < 0.0001. CDK9, one-way ANOVA, post-hoc Tukey test, F (3,20) = 27.98, *p* < 0.0001. pRNAPII, one-way ANOVA, post-hoc Tukey test, F (3,20) = 32,00, *p* < 0.0001. mGluR5 protein, one-way ANOVA, post-hoc Tukey test, F (3,20) = 21.31, *p* < 0.0001. ** *p* < 0.01 vs. Sham 21D. ## *p* < 0.01 vs. SNL 21D. mRNA, *n* = 5. protein, *n* = 6. (**g**) ChIP-qPCR assay of dorsal horn samples dissected at day 21 post operation. Compared to the sham operation, SNL decreased the levels of Hes1 antibody-precipitated but increased that of CDK9 and pRNAPII antibodies-precipitated exon 1a and exon 1b promoter fragments of mGluR5. These effects were all reversed by administering SNL animals with CDK9-targeting antisense siRNA, but not with the missense siRNA. Hes1, exon 1a, one-way ANOVA, post-hoc Tukey test, F (3,16) = 380.8, *p* < 0.0001. Hes1, exon 1b, one-way ANOVA, post-hoc Tukey test, F (3,16) = 29.54, *p* < 0.0001. CDK9, exon 1a, one-way ANOVA, post-hoc Tukey test, F(3,16) = 727.1, *p* < 0.0001. CDK9, exon 1b, one-way ANOVA, post-hoc Tukey test, F (3,16) = 856.4, *p* < 0.0001. pRNAPII, exon 1a, one-way ANOVA, post-hoc Tukey test, F (3,16) = 1888 *p* < 0.0001. pRNAPII, exon 1b, one-way ANOVA, post-hoc Tukey test, F(3,16) = 129.1 *p* < 0.0001. ** *p* < 0.01 vs. Sham 21D. # *p* < 0.05, ## *p* < 0.01 vs. SNL 21D. *n* = 5. (**h**) Immunofluorescence images of spinal slices dissected at day 21 post operation. When compared with the sham operation (left), SNL (middle) decreased the number and distribution of Hes1-positive (red) but increased that of CDK9- (green) and pRNAPII-positive (blue) neurons in the dorsal horn ipsilateral to operation; these effects were all reversed by administering SNL animals with CDK9 siRNA (SNL 21D + CDK9 siRNA) (right). Dashed lines indicate the margin of the dorsal horn. The images at the bottom are amplifications of the marked area, with arrows indicating the immunopositive neurons. Scale bar = 50 μm; Thickness = 50 μm.

Finally, the hierarchy and interactions among Hes1, CDK9, pRNAPII, and mGluR5 in the spinal mechanism underlying neuropathic allodynia development were examined by focal knockdown of spinal CDK9 expression using daily intrathecal administration of specific antisense siRNA to naïve rats. After confirming our protocol effectively decreased spinal CDK9 expression (1, 3, 5 μg, 10 μL; Figure 6a; 0.29 ± 0.03, 0.17 ± 0.02, and 0.11 ± 0.02) but affected neither the motor performance in naïve animals (5 μg, 10 μL; Figure 6b) nor the withdrawal threshold of sham-operated rats (5 μg, 10 μL; Figure 6c), CDK9 siRNA (5 μg, 10 μL) ameliorated SNL-induced allodynia, as evidenced by increments in the withdrawal threshold measured at days 19, 20, and 21 after operation (5 μg, 10 μL; Figure 6d; 4.17 ± 0.57 g, 6.74 ± 0.71, and 7.72 ± 1.15 g). Moreover, CDK9 siRNA (5 μg, 10 μL) significantly reversed the SNL-enhanced levels of mGluR5 mRNA as well as CDK9, pRNAPII, and mGluR5 proteins (Figure 6e,f; 1.11 ± 0.08, 0.39 ± 0.03, 0.46 ± 0.03, and 0.31 ± 0.03), yet, it unexpectedly increased the abundance of Hes1 (Figure 6f; 0.50 ± 0.05) in dorsal horn samples. Results of ChIP-qPCR demonstrated that SNL decreased the amount of Hes1antibody-precipitated but increased that of CDK9 and pRNAPII antibodies-precipitated mGluR5 promoter (exon 1a and exon 1b) in the dorsal horn sample; these effects were attenuated in SNL animals by daily administration of CDK9 siRNA (5 μg, 10 μL; Figure 6g; Hes1, 0.37 ± 0.00 and 0.42 ± 0.02; CDK9, 1.25 ± 0.03 and 0.60 ± 0.02; pRNAPII, 3.63 ± 0.03 and 1.54 ± 0.11). Consistently, SNL decreased the Hes1-positive but increased the pRNAPII- and CDK9-positive neurons in the dorsal horn of spinal slices; these effects were attenuated by daily administration of CDK9 siRNA (5 μg, 10 μL; Figure 6h). In summary, our results demonstrated that neuropathic insults provoke reciprocal Hes1-CDK9 expression that subsequently impacts CDK9 coupling/RNAPII phosphorylation-dependent transcription of mGluR5 to underlie allodynia development.

## 3. Discussion

The data in the current study demonstrated the involvement of signal-induced recruitment of CDK9 and subsequent RNAPII phosphorylation, a well-recognized mechanism mediating gene elongation to initiate the transcription cycle, as a novel pathogenic pathway for the development of neuropathic pain by modifying glutamatergic mGluR5 transcription and expression in the dorsal horn. In addition, by demonstrating that SNL downregulated spinal Hes1 expression that diminished its suppression of the recruitment of CDK9 and RNAPII as well as the subsequent phosphorylation of RNAPII on the mGluR5 promoter segments, these data for the first time demonstrated that Hes1, a transcription repressor, contributes to neuropathic pain development by impacting the spinal CDK9/pRNAPII-dependent mGluR5 transcription. Results in the current study defined Hes1 as a transcription repressor, whose impacts on synaptic plasticity are essential for the neural processing of pain by hampering signal-induced recruitment of CDK9/RNAPII-dependent gene transcription. These results provide a novel insight into the spinal mechanism leading to pain hypersensitivity, and thereby, raise a possible strategy to develop a medical treatment for neuropathic pain relief, which remains a challenge in clinical scenarios, by targeting the pre-transcription initiation steps of gene cycles.

Hes is a family of transcription factors that regulate neuronal function and morphology [5]. Studies have linked Hes1, a member of the Hes1-7 family, to neural plasticity underlying learning and memory [6] and to the pathogenesis of Alzheimer’s disease [24]. Analogous to studies that reveal that pain-associated plasticity that occurs in spinal neurons shares similar characteristics with the plasticity underlying learning and/or memory in brain regions [10], results in the current study provide evidence supporting the role of Hes1 in the spinal plasticity underlying pain hypersensitivity following neuropathic injury. Data in this study are consistent with a study that investigated Hes1 mRNA expression following diabetic neuropathy [25]. Nevertheless, in addition to Hes1, the potential contribution of other Hes families to pain-associated plasticity in the spinal neuraxis needs further studies to be elucidated because a genome-wide mapping analysis of brain tissue dissected from post-mortem Huntington’s disease patients revealed that the hyper-methylation of Hes4 promoter sequences was strikingly correlated with the measure of striatal degeneration and age-of-onset [26].

At the start of the transcription cycle, the positive transcriptional elongation factor (*p*-TEFb), which is comprised of cyclin T1 and CDK9, is recruited to and hence phosphorylates RNAPII on the promoter sequence of specific genes to facilitate gene elongation [27]. Data in the current study provide support for the role of the CDK9/pRNAPII cascade as a novel pathogenic pathway for the progression of spinal plasticity underlying neuropathic pain development by up-regulating transcription of mGluR5 in the dorsal horn. In addition to linking CDK9/pRNAPII-dependent mGluR5 transcription to the spinal plasticity mediating allodynia caused by neuropathic insults, the results in this study demonstrated that SNL-induced spinal CDK9/pRNAPII expression and coupling to the mGluR5 promoter, as well as mGluR5 transcription in the dorsal horn, were all reversed by focal overexpression of spinal Hes1. Together with our observation that neuropathic injury down-regulated spinal Hes1 expression, these data suggest that by hampering CDK9/RNAPII-regulated mGluR5 transcription, Hes1 acts as a suppressor to regulate the spinal plasticity underlying neuropathic pain development. This proposal is further supported by the fact that focal knockdown of Hes1 expression in naïve animals provoked CDK9 expression, RNAPII phosphorylation, coupling of CDK9 and pRNAPII to the mGluR5 promoter, and mGluR5 transcription in the dorsal horn, which resembles the sequelae caused by experimental neuropathic injury. Results in the current study are consistent with studies on immune cells that demonstrated that Hes1 selectively inhibits signal-induced recruitment of CDK9 that prevents RNAPII phosphorylation to impede transcription elongation of genes encoding chemokine, crucial for neutrophils’ chemotaxis [15]. Moreover, as such a mechanism widely works in various systems/organs, and pain-associated spinal plasticity shares molecular mechanisms with forms of plasticity occurring in brain regions, the potential involvement of Hes1-diminished CDK9/RNAPII-dependent transcription elongation in plasticity underlying brain functions, such as learning, memory, anxiety, and stress, is a really interesting issue that needs to be studied.

It is noteworthy, in this study, that focal knockdown of Hes1 expression resulted in an enhanced abundance of CDK9, and vice versa, i.e., a genetically reduced CDK9 level is accompanied with increased Hes1 expression in the dorsal horn of naïve rats, results that revealed a reciprocal change between the amount of spinal Hes1 and CDK9. Such a reciprocal interaction was also evidenced by observations that in the dorsal horn, SNL decreased Hes1 associated with enhancement of CDK9 expression; and lentivirus-induced Hes1 overexpression conversely reversed the SNL-reduced Hes1 expression and led to decreased CDK9 levels. Results in this study are somewhat different from that obtained from immune cells, in which knockout of Hes1 expression in macrophages did not globally alter cellular CDK9 levels [15]. Although the detailed mechanisms involved remain unclear, several potential causes might underlie this discrepancy. First, the samples explored were mainly in the dorsal horn, whereas their study investigated bone marrow-derived macrophages in culture; whether Hes1 exhibits diverse interactions with CDK9 in different cell types is unclear. Second, while focally knocked down/overexpressed spinal Hes1 expression was restricted to specific lumbar segments in the current study, their experiments generated knockout mice in which Hes1 expression was systemically deficient. The possibility that in the present in vivo preparations, Hes1 synthesized from organs/systems other than the dorsal horn could alter spinal Hes1–CDK9 interactions cannot be excluded, for such a systemic effect does not occur in cultures of homogenous cells that have no unnecessary impacts coming from other organs/systems. Moreover, in contrast to their study, which assayed Hes1-repressed chemokine transcription by treating macrophages with lipopolysaccharides, the current study investigated the impact of neuropathic insults on the Hes1-suppressed receptor transcription. Whether Hes1–CDK9 interactions vary when encountering different challenges, or alternatively, a reciprocal Hes1–CDK9 relationship occurs specifically in the nervous system when facing neural damage needs further studies to be elucidated because peripheral nerve injury in rats was demonstrated to downregulate ZAS3, a zinc finger protein, accompanied with reciprocal transcription of NF-kappa B-dependent genes in the dorsal root ganglion [28]. In addition, emerging evidence has demonstrated that spinal mGluR5 is a key mediator of neuroplasticity underlying pain hypersensitivity [29,30], and previous laboratory has reported that experimental neuropathic injury provoked behavioral allodynia along with enhanced dorsal horn mGluR5 expression, and conversely, administering neuropathic animals with an mGluR5-specific antagonist ameliorated behavioral allodynia [19,31]. The obtained results demonstrated that Hes1 contributes to pain-associated spinal plasticity through enhancement of mGluR5 transcription in the dorsal horn. However, a study show that restoring endocannabinoid signaling allows mGluR5 activation to increase infralimbic output and hence inhibit pain behaviors [32]. Nevertheless, the clear-cut mechanism in the spinal cord underlying this discrepancy needs further investigation.

## 4. Material and Methods

### 4.1. Neuropathic Pain Rat Model

A total of 429 male Sprague-Dawley rats weighing 200 to 250 g (age: 7–8 weeks) were used in the study; and rats were subjected to spinal nerve ligation (SNL) and sham operation, respectively. Animal procedures in this study were reviewed and approved by the Institutional Review Board of Taipei Medical University, Taipei, Taiwan (LAC-2017-0385) and Biosafety Committee, Mackay Medical College, New Taipei, Taiwan (B1060014). All animal experimental procedures were performed under the guidelines of the International Association for the Study of Pain [33]. In each group, 7 rats were used for the behavioral tests; 6 rats were used for Western blotting; 5 rats were used for the quantitative reverse-transcription PCR (RT-PCR), chromatin immunoprecipitation-qPCR (ChIP), and immunohistochemistry. The investigators in this study were blinded to the treatment groups. Five rats that showed neurological deficits after implantation of an intrathecal catheter were excluded from the statistical analysis.

Spinal nerve ligation (SNL) was used as the neuropathic pain model in rats [22,34,35]. Animals were anesthetized with isoflurane (induction, 5%; maintenance, 2% in room air), and the left L5–L6 spinal nerves were isolated and ligated tightly with 6-0 silk suture. Age-matched sham-treated control rats were used as the control group, the surgical procedures were identical to the nerve-ligated animals, except that the nerves were not ligated.

### 4.2. Intrathecal Catheter Implantation

To implant an intrathecal catheter, rats were anesthetized with isoflurane (induction, 5%; maintenance, 2% in room air) and inserted PE-10 tubing the dorsal aspect of lumbar enlargement of the rats’ spinal cord, as described previously [34]. Then, the outer part of the catheter was plugged and immobilized into the skin on closure of the incision made. After 3 days of recovery, rats displaying signs of neurological dysfunction were discarded and excluded from further experiments.

### 4.3. Behavioral Studies

To quantify tactile allodynia, rats were placed in individual plastic boxes on a mesh floor and habituated for 1 h. A series of calibrated von Frey filaments (Stoelting, Wood Dale, IL) were then applied to the plantar surface of the hind paw of animals with sufficient force to bend the filaments for 5 s to measure the paw-withdrawal threshold (up-down method) [21]. Motor coordination was evaluated in rats by the rota-rod apparatus (Panlab Harvard Apparatus, Barcelona, Spain). Rats were trained to three training trials at 3 to 4 h intervals on 2 separate days. The rod was set to accelerate from 4 to 30 rpm over a 180 s period. Three measurements were obtained at intervals of 5 min and were averaged for each test.

### 4.4. Western Blot Analysis

Rats’ dorsal horn (L4–5) samples were immediately removed, dissected, and homogenized in 25 mM Tris-HCl, 150 mM NaCl, 1% NP-40, 1% sodium deoxycholate, and 0.1% SDS with a complete protease inhibitor mixture (Roche). Samples were then put on ice for 1 h with shaking. The lysates were centrifuged at 14,000 rpm for 20 min at 4 °C. The supernatant was carefully collected, and the protein concentration was measured using a BCA protein assay reagent kit (Pierce, Rockford, IL, USA). Identical amounts of samples were loaded and separated by SDS-PAGE and electrophoretically transferred to PVDF membranes. The membranes were blocked with 5% nonfat milk or BSA in TBS containing 0.1% Tween-20 for 1 h and then incubated with one of the following primary antibodies at 4 °C for 1 h. The primary antibodies were as follows: Anti-Hes1 (rabbit, 1:1000, Genetex, Irvine, CA, USA), anti-mGluR5 (rabbit, 1:1000, Millipore, Billerica, Massachusetts), anti-CDK9 (rabbit, 1:1000, Cell Signaling, Beverly, MA, USA), anti-pRNAPII (rabbit, 1:1000, Abcam, Cambridge, USA), and anti-GAPDH (mouse, 1:2000, Santa Cruz Biotechnology, Santa Cruz, CA, USA). The membranes were then washed and incubated with peroxidase-conjugated goat anti-rabbit IgG (1:8000, Jackson ImmunoResearch, West Grove, PA, USA) or goat anti-mouse IgG (1:8000, Jackson ImmunoResearch) for 1 h at room temperature. The protein bands were visualized with an enhanced chemiluminescence detection kit (ECL Plus, Millipore) and then subjected to densitometric analysis using Science Lab 2003 software (Fuji, Tokyo, Japan). The protein band intensity was quantified by Images J.

### 4.5. Immunofluorescence Analysis

To test the specificity of the antibody used in the immunofluorescence, the molecular weight of the antibody was first checked using western blot analysis. In addition, the specificity of the antibody was further confirmed by the western blot analysis of samples treated with siRNA targeting Hes1 and CDK9 [16] and pRNAPII-selective inhibitor [16]. Rats were sacrificed and perfused intracardially with PBS followed by 4% paraformaldehyde in PBS (pH 7.4). The spinal cord samples were removed, post-fixed in the same fixative at 4 °C for 4 h, and cryoprotected in 30% sucrose in PBS overnight at 4 °C. The tissues were cut to a 30-µm thickness using a cryostat and were mounted on glass slides. The sections were washed and incubated with 5% BSA in PBS to block nonspecific binding. The sections were then incubated with rabbit anti-Hes1 (1:400, Genetex, Irvine, CA, USA) overnight (4 °C). Subsequently, the sections were rinsed in PBS and incubated with goat/donkey secondary antibodies conjugated to Alexa Fluor 488 (1:1500, Invitrogen) and Alexa Fluor 594 (1:1500, Invitrogen) were used (1 h, 37 °C). A Mix-n-Stain antibody labeling kit (Biotium, Hayward, CA, USA) were used to examine the interaction between Hes1 (1:400, Genetex, Irvine, CA, USA), CDK9 (rabbit, 1:1000, Cell Signaling, Beverly, MA, USA), and pRNAPII (rabbit, 1:400, Abcam, Cambridge, USA). Finally, these sections were rinsed in PBS, mounted on slides, dried, and cover-slipped. The sections were detected by a camera-coupled device (X-plorer; Diagnostic Instruments, Inc., USA) through a fluorescent microscope (LEICA DM2500, Germany).

### 4.6. Chromatin Immunoprecipitation-qPCR

Chromatin immunoprecipitation (ChIP) assays were performed with a commercial ChIP Kit (Millipore, Billerica, Massachusetts). Fresh dorsal horn samples were rapidly removed from anesthetized animals and cut into small pieces (1–2 mm^3^) using razor blades. The samples were then incubated in fresh 1% paraformaldehyde in PBS buffer, and gently agitated (10 min) at room temperature to crosslink proteins to DNA. After being washed, the samples were incubated in lysis buffer on ice. Subsequently, the tissues were sheared by sonication to generate chromatin fragments with an average length of 200 to 1000 base pairs. In total, 1% of the sonicated chromatin was saved as an input control for qPCR. The chromatin was pulled down for 2 h at room temperature using the following antibodies: Rabbit anti-Hes1 (rabbit, 1:1000, Genetex, Irvine, CA, USA), anti-CDK9 (rabbit, 1:1000, Cell Signaling, Beverly, MA, USA), pRNAPII (rabbit, 1:1000, Abcam, Cambridge, USA), or an equivalent amount of control IgG. Using protein G magnetic beads at 4 °C for overnight, the protein–DNA immunocomplexes were precipitated. After the beads were washed, they were re-suspended in ChIP elution buffer, incubated with proteinase K at 62 °C for 2 h, and then incubated at 95 °C for 10 min to reverse the crosslinks between the protein and DNA. ChIP signals were quantified by quantitative PCR analysis with a QuantStudio 3 Real-Time PCR System (Thermo Fisher Scientific, Waltham, USA). The specific primer pairs for the mGluR5 promoter region are described below.

Exon 1a: 5′-CATCTCTGGGTGGGAATGAG-3′ and 5′-GCTGGGCTGGCTCCTACT-3′.

Exon 1b: 5′-GGGTTAGGGAGGGAAGAGAA-3′ and 5′- GTGTGCACCATTTCAGCATC-3′.

### 4.7. Quantitative Reverse-Transcription PCR

The dissected dorsal horn (L4–5) samples were rapidly isolated in RNase-free conditions. Total RNA were extracted using RNA isolation kits (74106; Qiagen, Valenica, CA, USA). Total RNAs were reverse transcribed using complementary DNA reverse transcription kits (205311; Qiagen, Valenica, CA, USA).

Real-time PCR was performed on a QuantStudio 3 Real-Time PCR System (Thermo Fisher Scientific, Waltham, MA, USA). TaqMan Universal PCR Master Mix (2X) and TaqMan gene expression assay probes were GAPDH (Rn99999916_s1, Applied Biosystems, Carlsbad, CA, USA) and mGluR5 (Rn.PT.58.36061021, IDT, Coralville, IA, USA). Reactions (total volume, 20 uL) were comprised by incubation at 95 °C for 20 s, followed by 40 cycles of 1 s at 95 °C and 20 s at 60 °C, followed by a DNA melting curve for the determination of amplicon specificity. The expression level of the target mRNA was normalized to expression of GAPDH mRNA and analyzed by the standard 2 ^−△△*C*T^ method [36].

### 4.8. Injection of Lentiviral-Associated Vector

Rat lentiviral vectors expressing Hes1 (NM_024360, Hes1-2A-GFP, Hes1 LV) and the control (GFP alone, Control LV) were obtained from a medical supplier (Applied Biological Materials Inc., Richmond, BC, Canada). The rats received a single injection of 15 µL of lentiviral vector expressing Hes1 (8.6 × 10^7^ IU/mL) or control vector (2.2 × 10^7^ IU/mL) using an intrathecal catheter.

### 4.9. Small-Interfering RNA

The small-interfering RNA (siRNA) duplex used for Hes1 was 5′-CCAAUUUGCUUUCCUCAUC-3′, and the missense nucleotide sequence was 5′-UGAUAUUACCCUGAAUAUG-3′. The Hes1-specific siRNA and the missense siRNA were administered intrathecally for 4 consecutive days in animal rats (daily for 4 days in naïve rats or daily from days 3 to 6 after SNL). For the treatment siRNA, rats were anesthetized with isoflurane (induction, 5%; maintenance, 2% in room air).

### 4.10. Statistical Analysis

All data are expressed as the mean ± SEM. Data in this study were analyzed using SigmaPlot 10.0 (Systat Software) or Prism 6.0 (GraphPad). Paired two-tailed Student’s t-test was used to compare the means of groups. One-way ANOVA was used followed by Tukey’s post-hoc test to compare more than two groups. Two-way ANOVA followed by Tukey’s post-hoc test was used to assess changes in values for serial measurements over time. In all analyses, significance was set at *p* < 0.05.

## 5. Conclusions

By modifying glutamatergic mGluR5 transcription and expression in the dorsal horn, signal-induced recruitment of CDK9 and subsequent RNAPII phosphorylation contribute to a novel pathogenic pathway for the development of neuropathic pain. SNL downregulated spinal Hes1 expression and its suppression of the CDK9 and RNAPII recruitment and RNAPII phosphorylation on the mGluR5 promoter segments to mediate the development of neuropathic pain.

## Abbreviations

Hes 1	hairy and enhancer of split 1
CDK9	Cyclin dependent kinase-9
RNAPII	RNA polymerase II phosphorylation
mGluR5	metabotropic glutamate receptor subtype 5
SNL	Spinal nerve ligation
ChIP-qPCR	Chromatin immunoprecipitation-quantitative polymerase chain reaction
siRNA	small-interfering RNA
